# Genetic improvement of tocotrienol content enhances the oxidative stability of canola oil

**DOI:** 10.3389/fpls.2023.1247781

**Published:** 2023-09-18

**Authors:** Min Deng, Hao Chen, Wei Zhang, Edgar B. Cahoon, Yongming Zhou, Chunyu Zhang

**Affiliations:** ^1^ College of Agronomy, Hunan Agricultural University, Changsha, Hunan, China; ^2^ National Key Lab of Crop Genetic Improvement and College of Plant Science and Technology, Huazhong Agricultural University, Wuhan, China; ^3^ Center for Plant Science Innovation and Department of Biochemistry, University of Nebraska-Lincoln, Lincoln, NE, United States

**Keywords:** vitamin E, tocopherol, tocotrienol, *HvHGGT*, oxidative stability, canola

## Abstract

**Background:**

Tocotrienols and tocopherols, which are synthesized in plastids of plant cells with similar functionalities, comprise vitamin E to serve as a potent lipid-soluble antioxidant in plants. The synthesis of tocopherols involves the condensation of homogentisic acid (HGA) and phytyl diphosphate (PDP) under the catalysis of homogentisate phytyltransferase (HPT). Tocotrienol synthesis is initiated by the condensation of HGA and geranylgeranyl diphosphate (GGDP) mediated by homogentisate geranylgeranyl transferase (HGGT). As one of the most important oil crops, canola seed is regarded as an ideal plant to efficiently improve the production of vitamin E tocochromanols through genetic engineering approaches. However, only a modest increase in tocopherol content has been achieved in canola seed to date.

**Methods:**

In this study, we transformed barley *HGGT* (*HvHGGT*) into canola to improve total tocochromanol content in canola seeds.

**Results and discussion:**

The results showed that the total tocochromanol content in the transgenic canola seeds could be maximally increased by fourfold relative to that in wild-type canola seeds. Notably, no negative impact on important agronomic traits was observed in transgenic canola plants, indicating great application potential of the *HvHGGT* gene in enhancing tocochromanol content in canola in the future. Moreover, the oil extracted from the transgenic canola seeds exhibited significantly enhanced oxidative stability under high temperature in addition to the increase in total tocochromanol content, demonstrating multiple desirable properties of *HvHGGT*.

## Introduction

Oilseed rape (*Brassica napus* L.) is one of the most important oil crops worldwide. Currently, the seeds of most oilseed rape cultivars are of low erucic acid and glucosinolate contents, providing high-quality edible oil (canola oil) for human consumption, which contains approximately 55%–65% oleic acid (C18:1) (Zhao et al., 2019). In oilseed rape breeding, further enhancement of C18:1 content in seeds is an important goal because oleic acid possesses higher oxidization stability upon high-temperature frying than other unsaturated fatty acids enriched in oilseed rape seeds such as linoleic acid and linolenic acid (Romano et al., 2021). Another important breeding goal of oilseed rape is to genetically enhance the antioxidants in seeds, as previous studies have indicated that vegetable oils supplemented with extra antioxidants (such as tocopherol, phytosterol, butyl hydroxytoluol, ascorbic acid 6-palmitate, and some phytosterol fractions) have significant improvement of oxidative stability (Kamal-Eldin, 2006; Warner, 2007; Tabee et al., 2008).

Tocotrienols and tocopherols constitute vitamin E to serve as a potent lipid-soluble antioxidant in plants. They contain a polar chromanol head group derived from the shikimate pathway that binds to a structurally different hydrocarbon tail derived from a C_20_ isoprenoid. Tocopherols, which are widely present in leaves and seed embryos of plants (Cahoon et al., 2003; Horvath et al., 2006a; Horvath et al., 2006b; Falk and Munné-Bosch, 2010), are linked to a saturated hydrocarbon tail of phytyldiphosphate. Tocotrienols, which are the major form of vitamin E in the seed endosperm of most monocot species and some dicots such as Apiaceae (Horvath et al., 2006b; Falk and Munné-Bosch, 2010), are combined with a unsaturated hydrocarbon tail of geranylgeranyldiphosphate with three *trans* double bands. Within each class of vitamin E, four forms (α, β, γ, and δ) of tocopherols occur in plants, which differ in the number and position of methyl residues on the chromanol head group. α-Tocopherol, which contains three methyl groups on the chromanol ring and is widely present in the leaves and seed embryos of all plants, is considered to have the greatest nutritional value among different vitamin E forms because it is the most readily absorbed and retained in human and other mammalian cells (Kamal-Eldin and Appelqvist, 1996). In addition, γ- and δ-tocopherols, which are abundant in seed embryos containing two methyl groups on the chromanol ring but at different positions, have the greatest contribution to the oxidative stability of vegetable oils when exposed to prolonged higher temperature (Wagner and Elmadfa, 2000; Wagner et al., 2001; Warner et al., 2002), which is particularly important for the performance of vegetable oils in food processing and bio-based lubricants (Durrett et al., 2008).

**Figure 1:**
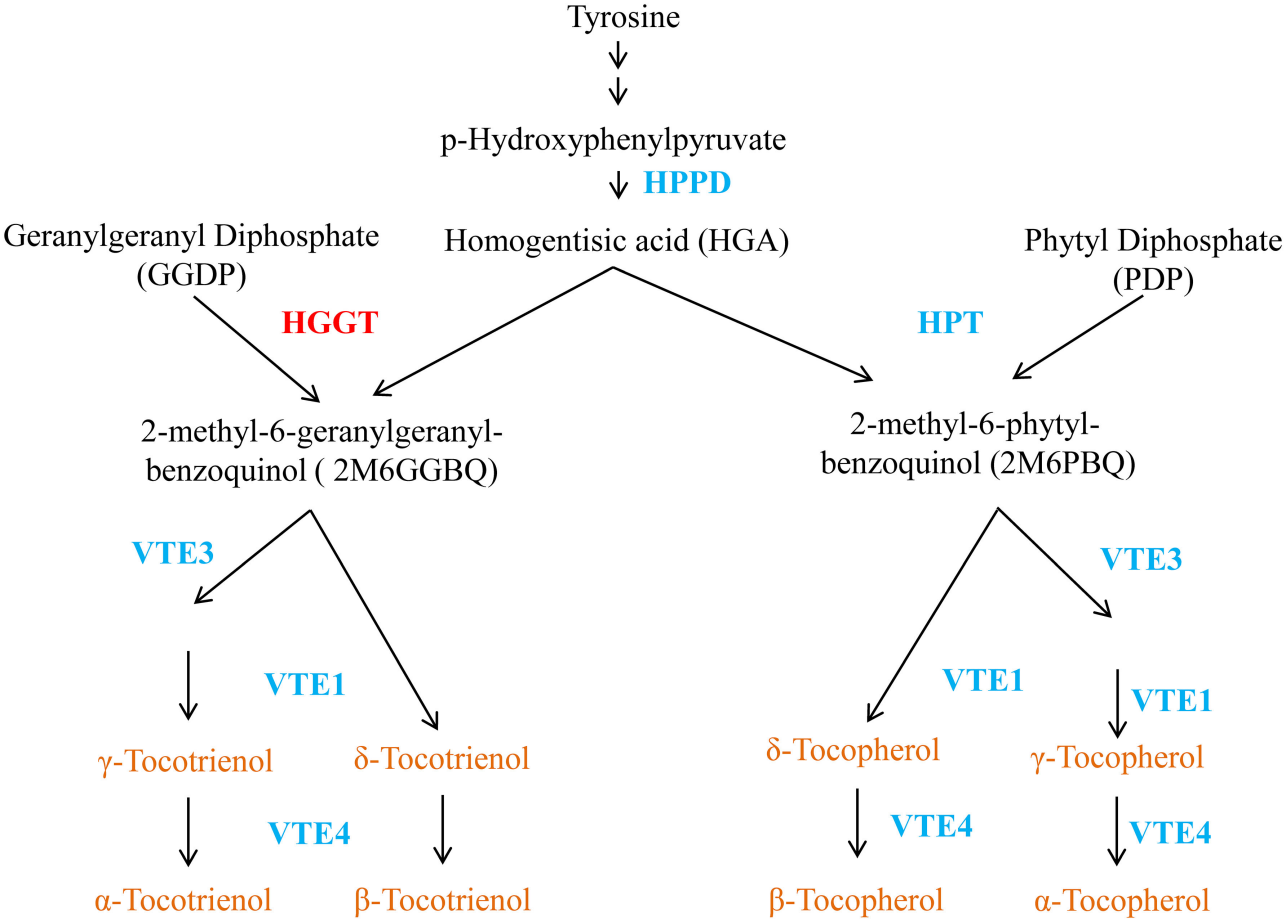
** **A vitamin E metabolic pathway in plant. HPPD, hydroxyphenylpyruvatedioxygenase; HGGT, homogentisate geranylgeranyl transferase; HPT, homogentisate phytyltransferase; HGA, homogentisic acid; VTE1, tocopherol/tocotrienol cyclase; VTE3, 2-methyl-6 phytyl/geranylgeranyl-benzoquinol methyltransferase; γ-TMT/VTE4, γ-tocopherol/tocotrienol methyltransferase.

Tocopherols and tocotrienols are synthesized in plastids of plant cells. The condensation of HGA from the shikimate pathway and phytyldiphosphate is a key step for the synthesis of tocopherols (**Figure 1**), which is catalyzed by homogentisate phytyltransferase (HPT) (Collakova and DellaPenna, 2001; Schledz et al., 2001; Savidge et al., 2002). The synthesis of tocotrienols differs from that of tocopherols only in the use of GGDP, rather than PDP, as the isoprenoid substrate for the initial condensation reaction. Previously, we have shown that this reaction is catalyzed by an enzyme designated as homogentisate geranylgeranyl transferase (HGGT) in monocot endosperm related to HPT, but has distinct substrate specificity with GGDP and PDP (Cahoon et al., 2003; Yang et al., 2011b). With the exception of HGGT for tocotrienol and HPT for tocopherol biosynthesis, the two biosynthetic pathways of tocochromanols share the same enzymatic system in the plastid.

Due to the wider occurrence of tocopherols than tocotrienols in the nature as described above, considerable research has been targeted at increasing the tocopherol form of vitamin E in plants. For example, overexpression of HPT has been applied to enhance tocopherol content in plants, which has typically resulted in only modest increases in vitamin E content, particularly in oilseed crops such as soybean and canola (Karunanandaa et al., 2005). Some other research attempted to upregulate the expression of hydroxyphenylpyruvatedioxygenase (HPPD), which catalyzes the final step of HGA synthesis, to increase the availability of this substrate for tocopherol biosynthesis (Jefford and Cadby, 1981; Lindstedt and Rundgren, 1982). However, this approach only resulted in little to moderate increases in total tocopherols in a variety of plant species (Falk et al., 2003; Karunanandaa et al., 2005; Tsegaye et al., 2002). Therefore, co-overexpression of multiple rate-limiting genes in the pathway were applied to further improve the total tocopherol content in a variety of crop plants. Although the co-expression of HPT and TMT resulted in a sixfold increase in total tocopherol content in lettuce leaves (Li et al., 2011), the highest record reported so far in canola seeds is a 2.4-fold increase by co-overexpression of triple genes including HPPD, HPT, and TC (Raclaru et al., 2006). Additionally, co-overexpression of the yeast/bacterium prephenate dehydrogenase and *Arabidopsis* HPPD and HPT in a variety of plants resulted in a 15-fold increase in total vitamin E content in the leaves of different transgenic plants, but the majority of them are in the form of tocotrienols (Karunanandaa et al., 2005). It has been reported that this increase in tocotrienol content is mediated by HPT as proved in *Arabidopsis* (Zhang et al., 2013). By contrast, the expression of *HvHGGT* in *Arabidopsis* leaves and maize embryos resulted in 6- to 10-fold increases in total vitamin E tocochromanols, primarily in the form of tocotrienols (Cahoon et al., 2003). Moreover, HGA has been proven to be a limiting substrate in the synthetic pathway of tocotrienols under the catalysis of HvHGGT in *Arabidopsis* seeds, which could result in a 7.9-fold increase in total tocochromanols relative to wild-type seeds (Zhang et al., 2013). More interestingly, a recent report indicated that overexpression of a rate-limiting gene *HvHGGT* in soybean seeds led to 8- to 10-fold increases in total vitamin E tocochromanols, primarily in the form of tocotrienols (Konda et al., 2020). However, hetero-expression of rice HGGT in soybean seeds only achieved a limited increase in total tocochromanol content due to slight *de novo* accumulation of tocotrienols (Kim et al., 2010). Overall, genetic engineering aiming at increasing tocotrienols through the overexpression of *HvHGGT* is more successful than the biofortification of tocopherols.

In this study, we determined to what extent seed-specific overexpression of single *HvHGGT* gene could enhance the total tocochromanol content in canola seeds and whether an increase in total tocochromanol content could further improve the oxidative stability of canola oil, a typically more stable vegetable oil with abundant frying-resistant oleic acid. Moreover, we also examined the main agronomic traits of the transgenic canola plants to reveal their potential to be applied to edible oil industry in the future.

## Materials and methods

### Plant materials and growth conditions

*Brassica napus* plants were planted in the green house and isolated nursery field (Huazhong Agricultural University, Wuhan, China). The field trial and green house did not require any specific permits, as the nursery was set up for this type of studies.

### Plasmid construction and *Brassica napus* transformation

The binary plant vector Napin-HGGT was used for seed-specific overexpression of barley *HGGT* (*HvHGGT*) gene in *B. napus*, which was described in details in a previous report (Zhang et al., 2013). This vector contained an open reading frame of *HvHGGT* driven by a strong seed-specific promoter for the *B. napus napin* gene.

Hypocotyl explants of *B. napus* winter inbred line JIA572 were transformed by *Agrobacterium tumefaciens* strain GV3101 in this study. The seeds were surface sterilized with 75% ethanol for 1 min and rinsed once with sterile distilled water. Then, the seeds were sterilized using 0.1% HgCl_2_ with 0.1% Tween 20 for 15 min and then rinsed three times with sterile distilled water. Hypocotyls were grown in a control environment (darkness, 24 h, 24°C) for 5 d. Then, the hypocotyls were cut into 8–10-mm fragments under aseptic conditions. The collected *A. tumefaciens* (OD = 0.6–0.8) was re-suspended with dilution medium (MS (Sigma-Aldrich, St. Louis, MO, USA), 30.0 g/L sucrose, and100 μm/L acetosyringone). The explants infected by the dilution medium with bacteria for 15–20 min were transferred into the callus induction medium (MS, 30.0 g/L sucrose, 18.0 g/L mannitol, 1 mg/L 2,4-D, 0.3 mg/L KT, 30 μm/L STS, 300.0 mg/L timentin, 25.0 mg/L kanamycin, and 6.0 g/L agar) for 20 d of cultivation in a control environment (light, 15 h, 24°C; dark, 9 h, 24°C). Subsequently, the callus was cultivated on a differentiation medium (MS, 10.0 g/L glucose, 0.25 g/L xylose, 0.6 g/L MES, 2.0 mg/L ZT, 0.1 mg/L IAA, 300.0 mg/L timentin, 25.0 mg/L kanamycin, and 6.0 g/L agar), and cultured for 20 d until production of buds. Subsequently, the buds were moved to the rooting medium (MS, 10.0 g/L glucose, and 10.0 g/L agar) for 2–4 weeks of cultivation.

### PCR and Southern blot analysis

Genomic DNA was extracted from leaf tissues by using the cetyltrimethylammonium bromide (CTAB) method as previously described (Fan et al., 2010). PCR reaction was carried out according to standard protocols (Molecular Cloning). The primers used for different PCR reactions were the gene-specific primers (5′-TGAGGAAATCAGGGGAGATG-3′ and 5′-GAAAGCCAACTGGACCAAGA-3′), the NPTI/II (5′-GTGCCCTGAATGAACTGC-3′ and 5′-CAATATCACGGGTAGCCA-3′), and the BnActinF/R (5′-AGCTGGAGACGGCTAAGAG-3′ and 5′-GTTGGAAAGTGCTGAGGGA-3′). PCR for all putative independent transformants was used to confirm the success of gene transformation. The primers used for different PCR reactions were gene-specific NPTI/II and the BnActinF/R.

Copy number analysis of transgenic plants was performed by Southern blot using the *Npt* gene as the probe. Approximately 30 μg genomic DNA of each sample was digested with restriction endonuclease *EcoR*I (Fermentas, Burlington, ON, Canada), fragmented on 0.7% agarose gel, and transferred to nylon membranes. Hybridization was performed using ^32^P-labeled partial cDNA fragments of *Npt* gene as the probe. The standard protocols of labeled probes followed the manufacturer’s instructions (Promega, Madison, WI, USA). Prehybridization and hybridization were performed under 65°C stringent conditions (Yang et al., 2011a; Zhang et al., 2015).

### Expression analysis of transgenic plants

Total RNA was extracted from different tissues including leaves, flowers, seedcases, and seeds by using the Trizol reagent (leaves and flowers) (Invitrogen, Gaithersburg, MD, USA) and TRIplant reagent (seedcases and seeds) (Bioteke, Beijing, China). For RT-PCR, the first-strand cDNA was synthesized from 1.5 mg total RNA using the TransScript One-Step gDNA Removal and cDNA Synthesis SuperMix kit (TransGen, China). The standard protocols followed the manufacturer’s instructions. The primers used for semi-quantitative PCR reaction are gene-specific primers and the BnActinF/R.

### Vitamin E isolation and HPLC analysis

Vitamin E was extracted with methanol and toluene dichloride. Approximately 15 mg of mature seeds was grinded with 500 μl mixture of methanol and toluene dichloride (9: 1 v/v) in 10-ml glass tubes, followed by the addition of 1.5-ml mixture involved above. The glass tubes with grinded seeds and the mixture were placed on a swing bed for more than 1 h. The mixture with grinded seeds was centrifuged for 10 min. Then, the supernatant was taken by suction for high-performance liquid chromatography (HPLC) analysis.

### Evaluation of agronomic traits and fatty acid composition

The T1 generation transgenic lines and wild type were planted in 2011–2012 in the security field. The field experiment was carried out following a random complete block design with three replications for transgenic lines and six replications for wild type. Each line was planted in two rows with 10–12 plants per row, a distance of 21 cm between two plants within a row, and 30 cm between two rows. All materials were grown in the experimental farm of Huazhong Agriculture University, Wuhan, China.

The agronomic traits were measured for five to seven plants of each replication per line. The 1,000-seed weight (TSW), plant height (PH), main infloresence length (MIL), primary effective branch number (PBN), silique length (SL), and seeds per silique number (SSN) were evaluated. In addition, the fatty acid composition was analyzed by near-infrared reflectance spectroscopy (NIR).

### Oil stability analysis

Approximately 40 g of seeds from the parent and transgenic progeny lines was grinded into fine powders in a blender and extracted by heptane for 2 h, respectively, and then centrifuged at 1,000×*g* for 15 min to purify the heptane-dissolved oil from the powders of seed oil extraction residue. After drying under N_2_ gas, approximately 10 ml of each kind of classified oil was obtained and then sealed under N_2_ and kept at −20°C for long-term storage and further analysis. The oxidative stability index (OSI) of the extracted oils was measured following a previously described method (Yang et al., 2013). Due to the limitation of seed amount, two independent samples were measured for each type of oil.

## Results

### Generation and molecular characterization of canola plants transformed with *HvHGGT*

**Figure 2:**
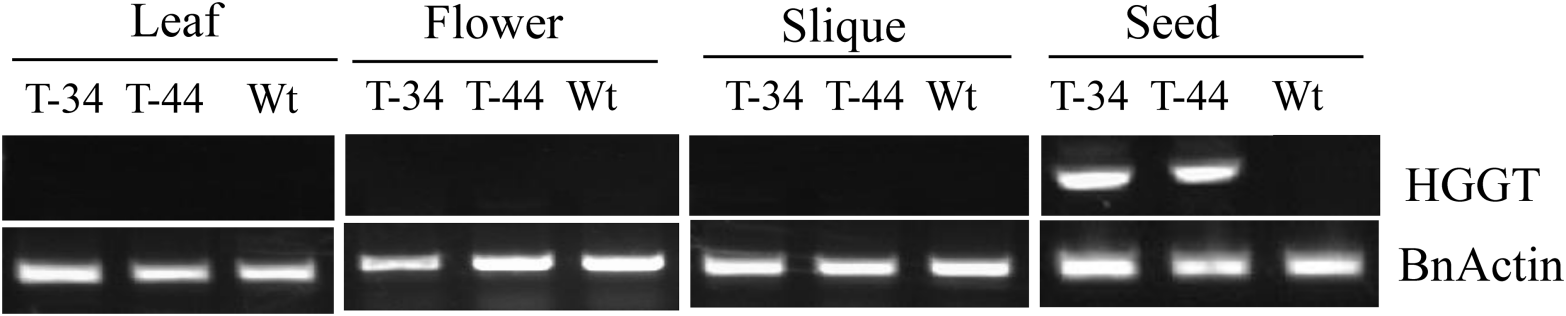
Semi-quantitative RT-PCR analysis of leaves, flowers, seedcases, and seeds from wild-type and transgenic plants.

Totally, 45 putative independent transformants were generated through *A. tumefaciens*-mediated transformation. Southern hybridization studies were performed on the transgenic plants, which were raised from 15 randomly selected PCR-positive (T0 generation) transformants along with non-transformed plants. Almost all plants showed multiple hybridization bands with the NPTII probe, suggesting multi-copy insertion (**Supplementary Figure S1**). To further confirm the tissue-specific expression pattern of the transgene, a semi-quantitative RT-PCR analysis was carried out with RNA extracted from four different tissues including leaves, flowers, siliques, and developing seeds from two transgenic lines and wild-type plants. As expected, the transcription of transgenic *HGGT* was only detectable in developing seeds (**Figure 2**).

### Profiling of tocochromanols in T1 seeds

**Figure 3:**
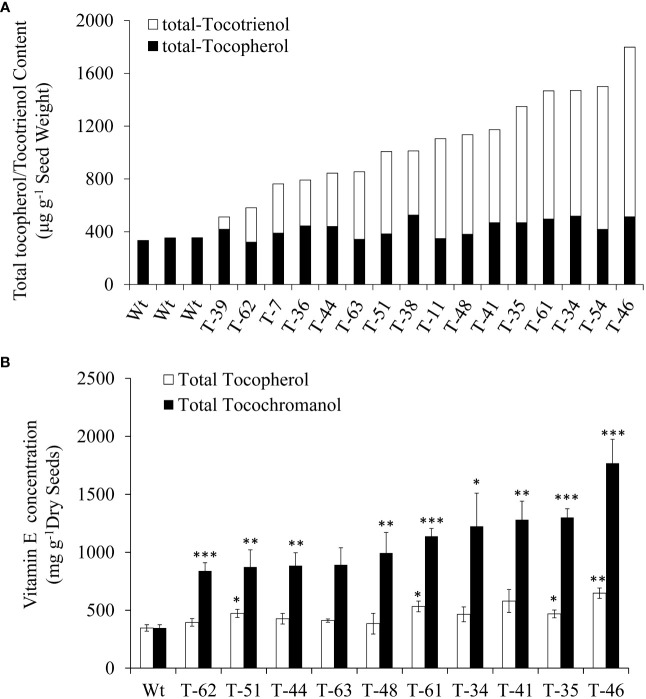
Total tocopherol and tocotrienol content of T1 **(A)** and T2 **(B)** generations canola transgenic events and wild type. The significance tests are calculated using the Student’s-t test. * , ** and *** indicate significance levels between the transgenic lines and the wild type of different traits at p<0.05, p<0.01 and . p<0.001 Values are shown in means ± standard error.

Subsequently, self-pollinated seeds from 16 T1 generation transgenic plants were employed for the profiling of tocochromanols by using HPLC. All the 16 transgenic plants showed increases in tocochromanols relative to the wild type, as 1,798.5 μg/g tocochromanols was detected in transgenic dry seeds from line 46, while only 346.9 μg/g tocochromanols was detected in wild-type dry seeds. The increases in the content of tocochromanols in transgenic lines ranged from 47.5% (164.8 μg/g) to 418.4% (1,451.6 μg/g) compared with that in wild type, with an average increase of 212.8% (738.1 μg/g) (**Figure 3A**). Moreover, a significant amount of tocochromanols in the form of tocotrienol was generated in transgenic seeds, which was hardly detected in wild-type seeds. In terms of tocotrienol content, it ranged from 93.0 μg/g dry seeds (line 39) to 1,285.3 μg/g dry seeds (line 46), with an average of 655.3 μg/g dry seeds for all transgenic lines (**Figure 3A**). This finding is consistent with our previous results obtained in *Arabidopsis* seeds (Zhang et al., 2013), suggesting that *Arabidopsis* and canola may be highly similar in vitamin E biosynthetic metabolism. In addition, all transgenic events resulted in slight to modest increases in the total tocopherol content compared with the wild-type seeds. Generally, the seeds with a higher content of tocotrienols accumulated relatively higher levels of tocopherols. This result is consistent with the previous finding in the transformation with the same *HvHGGT* gene from barley, demonstrating that *HvHGGT* can incorporate substrate PDP for tocopherol biosynthesis as reported by Yang et al. (2013) in *Arabidopsis*.

### Composition and total content of tocochromanols in T2 seeds

**Figure 4:**
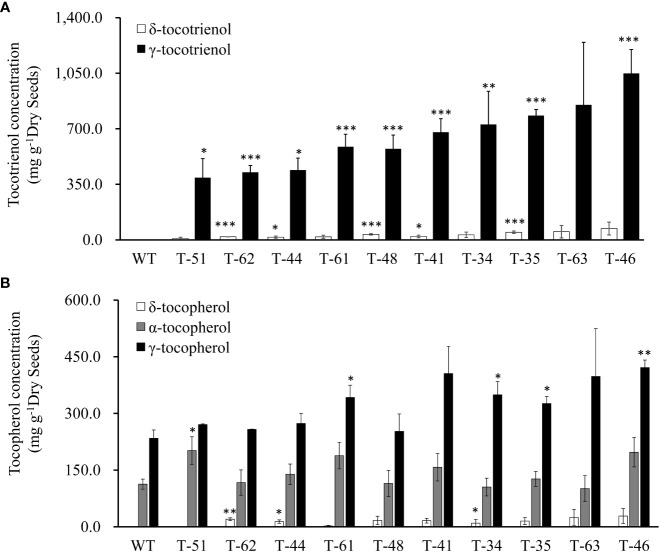
Vitamin E tocochromanols content of canola transgenic events and wild type. **(A)** δ-Tocotrienol and γ-tocotrienol content of T2 canola transgenic events and wild type. **(B)** δ-Tocopherol, α-tocopherol, and γ- tocopherol content of wild-type and T2 canola transgenic events. The significance tests are calculated using the Student’s-t test. * , ** and *** indicate significance levels between the transgenic lines and the wild type of different traits at p<0.05, p<0.01 and . p<0.001 Values are shown in means ± standard error.

To reveal the hereditary stability of *HvHGGT* transgene through generations, the total tocochromanol content in the seeds of 10 T2 generation transgenic lines was determined by HPLC. The results showed that T2 generation seeds had almost the same level of total tocochromanols (**Figure 3B**) as corresponding T1 generation seeds (**Figure 3**). However, highly divergent total amounts of tocochromanols were observed among three measurements of each T1 generation transgenic line, suggesting complex segregation of multiple copies of *HvHGGT* transgene in different seeds of T2 generation. As for the composition of tocochromanols, α-tocotrienol was undetectable in all transgenic events and wild-type seeds, whereas a small portion of δ-tocopherol was observed in almost all transgenic events but not in wild-type seeds. These results implied that the HvHGGT activity may contribute to the production of δ-tocopherol in transgenic seeds. Moreover, except for a small portion of δ-tocotrienol, the remaining tocotrienols were dominated by γ-tocotrienol in all transgenic seeds (**Figure 4**). The α-tocopherol to γ-tocopherol ratio ranged from 0.25 to 0.75 in transgenic seeds and was 0.48 in wild-type seeds, indicating significantly different efficiency of VTE4 activity in converting γ-tocopherol and γ-tocotrienol into α-tocopherol and α-tocotrienol, respectively.

### Oxidative stability of canola oil with enhanced tocochromanol content under high temperature

**Figure 5:**
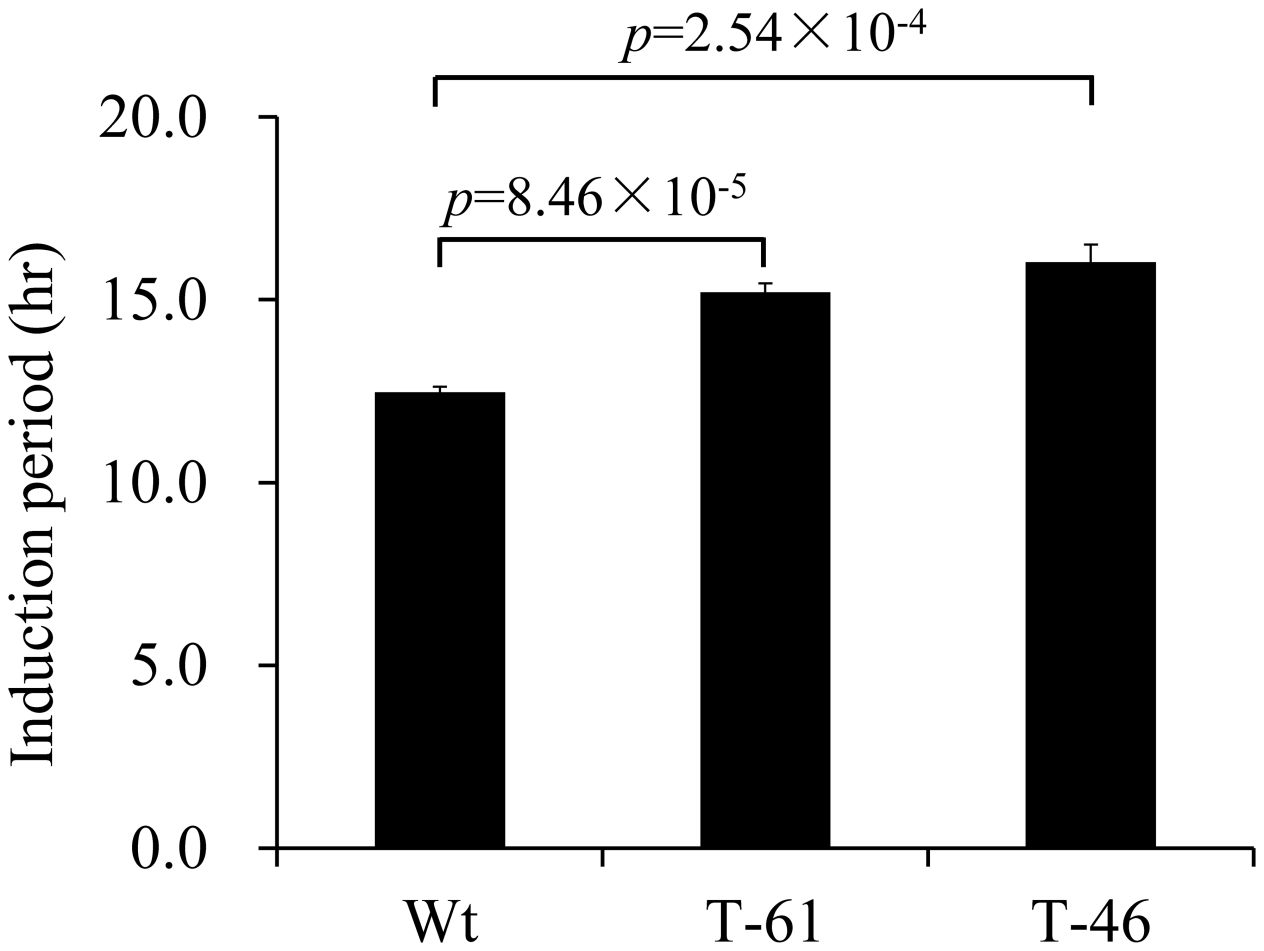
Oxidative stability index analysis of oil from canola events.

Tocochromanols such as δ- and γ-tocochromanols have been reported to confer strong oxidative stability to vegetable oil under high temperature (Wagner et al., 2001). To determine the oxidative stability of the fresh oil with enhanced total tocochromanol content from canola seeds overexpressing *HvHGGT*, T2 seeds from line 46 with the highest content of total tocochromanols and line 61 with the lowest content of total tocochromanols were used for oil extraction. Due to the limited amount of seeds, 3 ml fresh canola oil from each line with two replicates was applied for the test of oxidative stability with the apparatus of Rancimat under 110°C. The oxidative stability was represented by the induction period, with a higher value of induction period indicating stronger stability of the tested samples. As shown in **Figure 5**, compared with the oil extracted from wild-type seeds, the oil extracted from the seeds of lines 46 and 61 had 29% and 22.6% increases in induction period, respectively (**Figure 5**), demonstrating that enhancement of tocochromanol content can improve the oxidative stability of oil under extreme heating stress.

### Impact of seed-specific expression of *HvHGGT* on important agronomic traits

As demonstrated above, seed-specific overexpression of *HvHGGT* resulted in the highest increase in total tocochromanol content in seeds and stronger oxidative stability of oil. Moreover, it is highly necessary to further investigate how *HvHGGT* overexpression in canola seeds affects other important agronomic traits such as plant height, branch number per plant, seed number per pod, weight of thousand seeds, and traits of seed quality such as the composition and content of fatty acids because it is not worth to improve one agronomic trait at the expense of other important traits. The results obtained from field experiment indicated that *HvHGGT* overexpression has no significant negative impact on the above agronomic traits (**Table 1**). Moreover, there were no significant changes in fatty acid composition and content in the transgenic seeds compared with those of wild-type seeds, except for significantly high oil content in transgenic line 34 and 41 than the wild type (**Table 2**). Collectively, all these findings demonstrate great application potential of *HvHGGT* to the seed production of canola to enhance the total content of tocochromanols in the future.

**Table 1:** Agronomic traits in canola transgenic events and wild type.

	N	TSW (g)	PH (cm)	MIL (cm)	PBN	SL (cm)	SSN
T-34	19	3.76 ± 0.21	171.96 ± 3.78	59.40 ± 1.57	5.95 ± 0.62	4.40 ± 0.15	12.84 ± 1.28
T-35	17	3.27 ± 0.35	168.97 ± 6.28	56.35 ± 5.71	6.41 ± 0.17	4.34 ± 0.17	11.18 ± 0.38^*^
T-41	20	3.52 ± 0.21	166.31 ± 5.52	54.97 ± 3.70	6.93 ± 0.72	4.63 ± 0.20	13.39 ± 1.22
T-44	18	3.66 ± 0.14	167.20 ± 2.19	59.11 ± 1.29	5.32 ± 0.45	4.63 ± 0.15	13.44 ± 0.48
T-46	15	3.57 ± 0.42	165.85 ± 1.54	56.49 ± 2.10	5.34 ± 0.29	4.47 ± 0.13	11.95 ± 0.74
T-61	15	3.76 ± 0.21	161.94 ± 4.61	55.53 ± 2.78	5.33 ± 0.21	4.12 ± 0.18^*^	10.34 ± 0.17^**^
T-62	12	3.50 ± 0.24	150.56 ± 5.05^*^	55.42 ± 0.96^**^	6.47 ± 0.37	4.45 ± 0.01^*^	10.29 ± 1.12^*^
Wt	33	3.78 ± 0.10	163.24 ± 2.27	61.46 ± 1.23	5.89 ± 0.39	4.70 ± 0.10	13.59 ± 0.75

TSW, 1000-seed weight; PH, plant height; MIL, main infloresence; PBN, primary effective branch number; SL, silique length; SSN, seeds per silique number. The significance tests are calculated using the Student’s-t test. * and ** indicate significance levels between the transgenic lines and the wild type of different traits at p<0.05 and p<0.01. Values are shown in means ± standard error.

**Table 2:** Fatty acid composition (%) of seed lipids in canola transgenic events and wild type.

	C16:0	C18:0	C18:1	C18:2	C18:3	C20:1	C22:1	Oil content
T-34	5.20 ± 0.08	2.67 ± 0.29	63.32 ± 2.15	20.48 ± 1.82	6.84 ± 0.45	0.62 ± 0.04	0.87 ± 0.05	37.37 ± 0.10^*^
T-35	4.82 ± 0.13	2.97 ± 0.17	68.11 ± 0.16	17.63 ± 0.25	4.99 ± 0.12	0.57 ± 0.03	0.90 ± 0.02	34.84 ± 0.29
T-41	5.10 ± 0.17	2.99 ± 0.26	63.95 ± 0.95	19.66 ± 0.82	6.84 ± 0.52	0.63 ± 0.03	0.84 ± 0.00	36.91 ± 0.32^*^
T-44	4.91 ± 0.04	2.59 ± 0.14	65.82 ± 1.07	19.01 ± 0.88	6.24 ± 0.31	0.58 ± 0.05	0.84 ± 0.02	35.94 ± 0.28
T-46	5.23 ± 0.35	2.57 ± 0.17	60.10 ± 1.30^**^	23.37 ± 1.14^*^	7.25 ± 0.71	0.61 ± 0.09	0.87 ± 0.07	33.68 ± 0.05
T-61	5.88 ± 0.45	2.52 ± 0.19	64.27 ± 2.33	19.97 ± 1.99	5.91 ± 0.17	0.56 ± 0.05	0.89 ± 0.02	34.83 ± 0.04
T-62	5.25 ± 0.21	2.64 ± 0.11	68.93 ± 1.11	16.63 ± 0.92	5.10 ± 0.28	0.62 ± 0.02	0.84 ± 0.01	34.87 ± 0.33
Wt	5.46 ± 0.30	2.58 ± 0.11	66.86 ± 1.38	18.20 ± 1.05	5.48 ± 0.52	0.56 ± 0.06	0.85 ± 0.02	34.96 ± 0.77

The significance tests are calculated using the Student’s-t test. * and ** indicate significance levels between the transgenic lines and the wild type of different fatty acid composition at p<0.05 and p<0.01. Values are shown in means ± standard error.

## Discussion

In the present study, previously identified *HvHGGT* was employed for genetic transformation of canola (*B. napus*) to improve the total tocochromanol content in seeds. As expected, the total tocochromanol content was increased by an average of 2.1- and 3.4-fold in T1 and T2 generation transgenic canola seeds compared with that in untransformed seeds. Notably, this is one of the most successful studies to significantly improve the content of secondary metabolites by genetic engineering with a single gene in major crops. In contrast, the best reported performance was an approximately 2.4-fold increase in total tocopherol content in the seeds of transgenic canola with co-overexpression of multiple key genes in the pathway (Raclaru et al., 2006). Furthermore, it is supposed that the total tocopherol content can be further improved in canola seeds by combining the overexpression of *HvHGGT* and downregulation of HGA production, since homogentisate availability was found to be a limiting factor of HvHGGT-mediated tocochromanol production in *Arabidopsis* seeds (Zhang et al., 2013). In addition, the total vitamin E tocochromanols concentrations were ~10-fold higher than those detected in seeds from the non-transformed lines in soybean (Konda et al., 2020). However, to our knowledge, a significant enhancement of total tocochromanol content in *Arabidopsis* seeds would significantly reduce the seed longevity at room temperature (unpublished data). Therefore, considering that the functionality of tocotrienols is very similar to or stronger than that of tocopherols (Theriault et al., 1999; Sen et al., 2006; Aggarwal et al., 2010), the two- to fourfold increase in total tocochromanol content achieved in transgenic seeds relative to that of wild-type seeds in this study may be the most promising option for a better balance with other important agronomic traits. Our results showed that the change in total tocochromanol content in the seeds has no negative impact on the growth and yield of transgenic plants compared with the untransformed control plants, as verified by their highly similar performance in field traits, fatty acid composition, and total oil content. Collectively, all these results indicate a great potential of the *HvHGGT* gene to be applied for the efficient production of total tocochromanols in canola in the future. However, for future commercial production, transgenic plants with single insertion of *HvHGGT* are strongly required, and a marker-free technology instead of selection marker gene linking to the target gene is also needed.

The oxidative stability of vegetable oils is primarily determined by their vitamin E content and composition and by their degree of fatty acid unsaturation. As a demonstration of the importance of vitamin E, removal of tocopherols from vegetable oils has been shown to greatly reduce the oxidative performance of oils in frying application (Huang et al., 1995). Conversely, supplementation with tocopherols, particular those in the δ and γ forms, can improve the shelf-life and high-temperature oxidative stability of vegetable oils (Huang et al., 1995; Wagner et al., 2001; Isnardy et al., 2003). As is known, canola oil is desirable for its long shelf life and high stability under high temperature during deep frying, particularly due to its high content of oleic acid (C18:1) (Warner and Knowlton, 1997; Matthäus, 2006). Surprisingly, further increasing the total tocochromanol content in transgenic canola seeds could confer stronger oxidative stability to the extracted oil, as indicated by the extended induction period. This enhancement in oxidative stability can be mainly attributed to the additional increase in tocochromanols, particularly in the form of tocotrienol.

The conclusion of the present study is further confirmed by the result that there was no obvious difference in fatty acid profile between transgenic and wild-type seeds. In addition to delaying oil oxidation during food processing, tocotrienols and tocopherols, particularly in the δ- and γ-form, are also essential and active nutrients in human diet. However, this study showed that *HvHGGT* overexpression only resulted in limited increases in the amount of α-tocopherol and an undetectable amount of α-tocotrienol in transgenic canola seeds. To meet the demand for α-tocotrienol, in addition to HGGT, γ-TMT is also required to efficiently convert the dominant form of γ-tocotrienol into α-tocotrienol based on many reported successful examples (Van Eenennaam et al., 2003; Yusuf and Sarin, 2007; Li et al., 2011).

## Data availability statement

The original contributions presented in the study are included in the article/**Supplementary Material**. Further inquiries can be directed to the corresponding author.

## Author contributions

CZ and YZ designed and supervised this study. MD and HC collected the agronomic traits. MD and WZ detected the tocochromanols content in canola seed. MD and CZ performed the data analysis and prepared the manuscript. EC and YZ revised this manuscript. All authors contributed to the article and approved the submitted version.

## Supplementary material

Supplementary Figure 1 Southern blot analysis of wild type and transgenic plants.
