# Supplementary figures and images for: Genetic improvement of tocotrienol content enhances the oxidative stability of canola oil
Source: Front Plant Sci. 2023 Sep 18;14:1247781. doi: 10.3389/fpls.2023.1247781 (PMC10543761; doi:10.3389/fpls.2023.1247781)

T-7 T-11 T-34 T-35 T-36 T-38 T-39 T-41 T-44 T-46 T-48 T-51 T-54 T-61 T-63 Wt

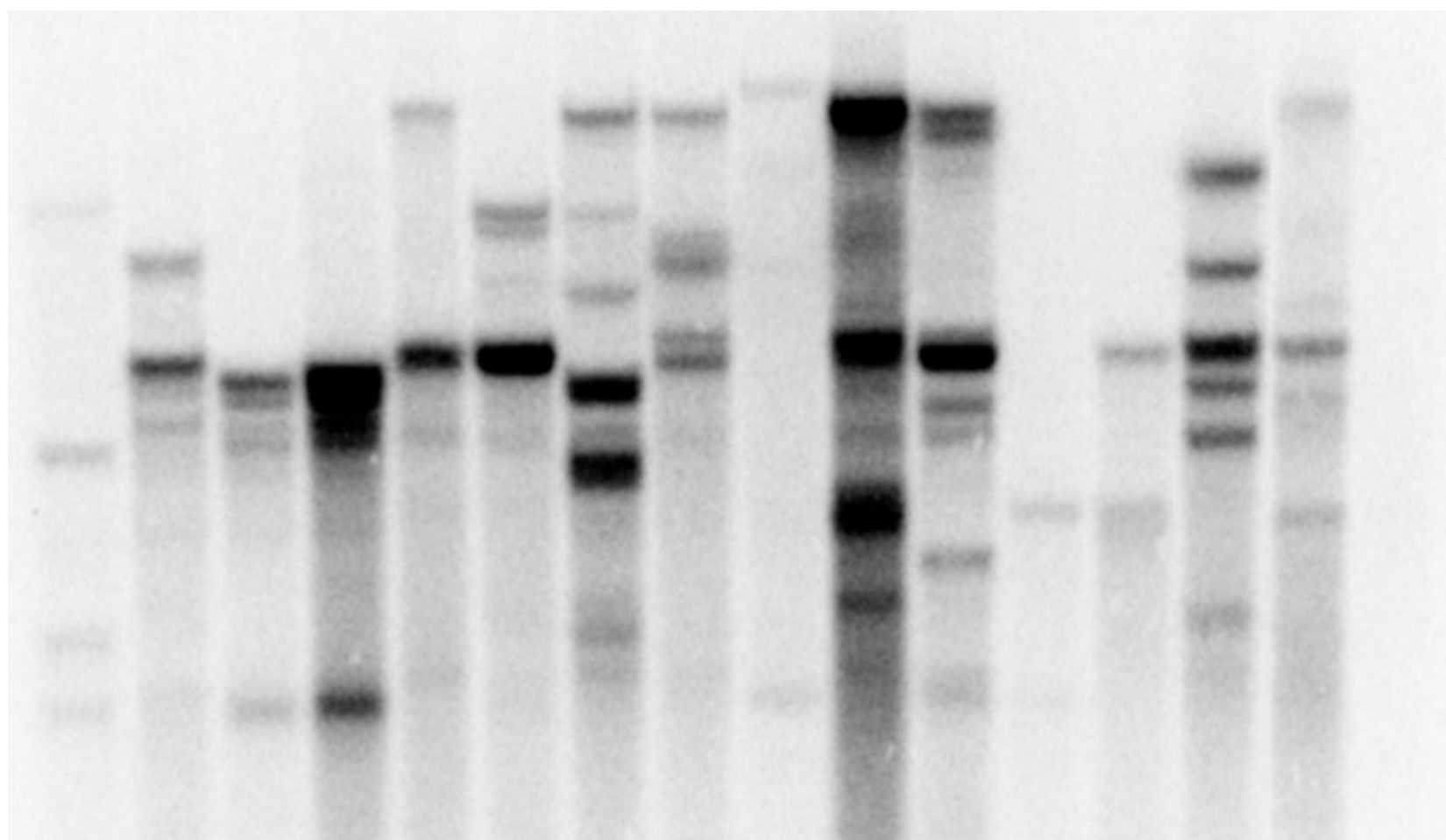

Figure 2. Southern blot analysis of wild type and transgenic plants.

Supplement: Supplementary Figure 1 — Southern blot analysis of wild type and transgenic plants. [file Image_1.pdf]
